# Daridorexant in severe obstructive sleep apnea: effects on sleep-disordered breathing and sleep

**DOI:** 10.1093/sleep/zsag145

**Published:** 2026-05-22

**Authors:** Ingo Fietze, Marie-Laure Boof, Katharina Lederer, Vincent Lemoine, Cedric Vaillant, Jean-Louis Pépin

**Affiliations:** Center of Sleep Medicine, Charite–Universitätsmedizin Berlin, Berlin, Germany; Idorsia Pharmaceuticals Ltd, Allschwil, Basel-Landschaft, Switzerland; Advanced Sleep Research GmbH, Berlin, Germany; Idorsia Pharmaceuticals Ltd, Allschwil, Basel-Landschaft, Switzerland; Idorsia Pharmaceuticals Ltd, Allschwil, Basel-Landschaft, Switzerland; HP2 Laboratory, INSERM U1300, Grenoble Alpes University Hospital and EFCR Laboratory, Thorax and Vessels Division, Grenoble, France

**Keywords:** daridorexant, dual orexin receptor antagonist, nighttime respiration, severe obstructive sleep apnea, sleep

## Abstract

**Study Objectives:**

To evaluate the effect of daridorexant on nighttime respiratory function and sleep in adults with severe obstructive sleep apnea (OSA) without insomnia.

**Materials and Methods:**

This randomized, double-blind, placebo-controlled, two-period, crossover trial was conducted at a single sleep center in 16 adults (≥18 years) with severe OSA without insomnia. In each period, daridorexant 50 mg or placebo was administered every evening for 5 days. Primary and secondary endpoints were the treatment differences (daridorexant–placebo) for apnea/hypopnea index (AHI) and oxygen saturation (SpO_2_) during total sleep time (TST), respectively, after last dosing. A mean increase in AHI ≥10 events/h and mean decrease in nocturnal SpO_2_ ≤–2% were the minimum changes considered to be clinically meaningful negative effects. Other endpoints included TST, latency to persistent sleep (LPS), and wake after sleep onset (WASO).

**Results:**

Mean baseline AHI was 51.2 events/h (range 30.8, 82.2) and mean SpO_2_ during TST was 92.1% (range 88.5, 94.3). No clinically meaningful effect of daridorexant on AHI or SpO_2_ during TST was detected. Treatment differences were −3.7 events/h (one-sided 95% CI ≤ +4.2) and − 0.12 % (one-sided 95% CI ≥ −0.6), respectively. Compared with placebo, daridorexant increased TST by 32.5 min (90% CI: 6.9, 58.2), associated with shorter LPS (−10.3 min [90% CI: −20.6, −0.02]) and a trend towards reduced WASO (−15.2 min [−31.2, 0.9]). Four adverse events were reported (daridorexant *n* = 3; placebo *n* = 1), all of mild intensity and none related to respiratory function.

**Conclusion:**

Short-term treatment with daridorexant does not impair sleep-disordered breathing and may improve sleep in patients with severe OSA.

**Clinical trial:**

ClinicalTrials.gov, https://clinicaltrials.gov/study/NCT05458193, NCT05458193.

**Statement of Significance**

Obstructive sleep apnea (OSA) is highly prevalent and associated, in 30%-50% of cases, with insomnia-related symptoms, yet the safety of insomnia medications in OSA remains unclear. Daridorexant, a dual orexin receptor antagonist for the treatment of adults with insomnia disorder, previously showed no negative effect on sleep-disordered breathing in participants with mild/moderate OSA. This randomized, double-blind, placebo-controlled, crossover trial evaluates daridorexant 50 mg (maximum therapeutic dose) in participants with severe OSA without insomnia. Repeated dosing (5 nights) did not impair nighttime respiratory function, as assessed by apnea/hypopnea index and nocturnal oxygen saturation. Moreover, improvements in sleep characteristics were observed with daridorexant, extending evidence that daridorexant 50 mg is safe and well-tolerated and may improve sleep in adults with severe OSA.

## Graphical Abstract

**Graphical Abstract:**
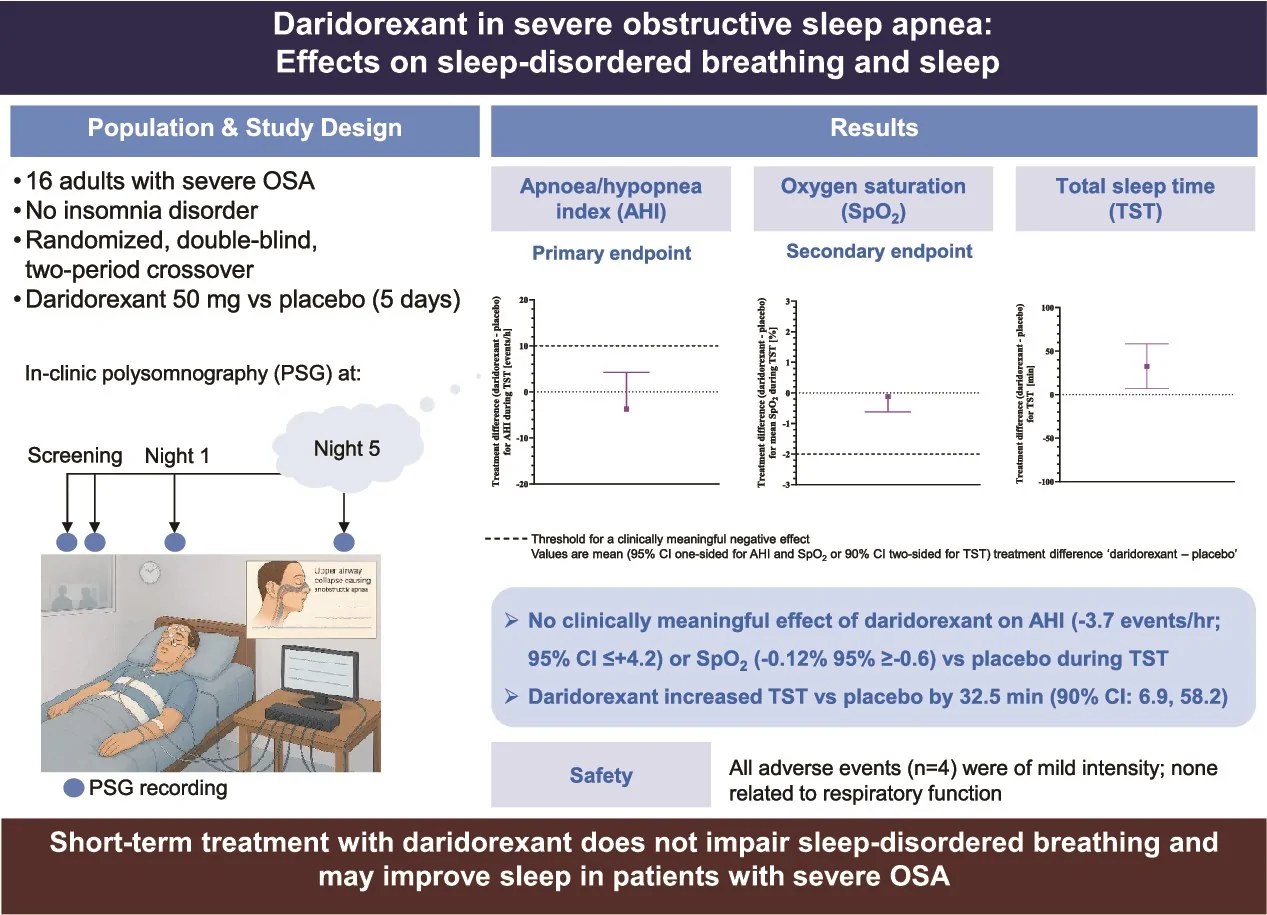

## Introduction

Obstructive sleep apnea (OSA) is a prevalent sleep-related breathing disorder characterized by repeated collapse and obstruction of the upper airway during sleep. These events lead to either a partial reduction in airflow (hypopnea) or a complete cessation of airflow (apnea), resulting in intermittent oxygen desaturation and frequent sleep disruptions due to arousals or full awakenings upon the resumption of ventilation [1].

The global burden of OSA is substantial and is projected to increase significantly over the next 30 years [2]. Current estimates suggest that approximately one billion adults aged 30–69 years worldwide may have OSA, including approximately 425 million individuals with moderate to severe OSA for whom treatment is generally recommended [3]. The true prevalence is likely higher. There is a well-documented linear relationship between age and OSA [4], and these estimates exclude adults over 70 years of age. Moreover, a substantial proportion of patients with OSA remain undiagnosed, largely due to the non-specific nature of symptoms and a general under-recognition by healthcare providers [5, 6].

OSA frequently coexists with insomnia [7, 8]. Approximately 30%-50% of adults with OSA report clinically significant insomnia symptoms [9]. This comorbidity complicates both diagnosis and management strategies [10]. Some studies suggest that the presence of insomnia symptoms can negatively affect treatment outcomes in patients with OSA, especially in patients using positive airway pressure therapies and mandibular advancement devices [11–14]. It is also unclear whether treatments for insomnia disorder are safe and effective in patients with OSA [15]. Given the high prevalence of comorbid insomnia disorder and the high rate of undiagnosed OSA, it is desirable to develop insomnia medications that do not have deleterious effects on respiratory function. It is therefore essential to study their effects on this function.

Daridorexant is a dual orexin receptor antagonist (DORA) approved for the treatment of adult patients with insomnia disorder [16]. Previous clinical trials have shown that administration of single and multiple doses of daridorexant 50 mg, the maximum therapeutic dose, has no negative effects on nighttime respiratory function in participants with mild or moderate OSA [17, 18] or with moderate chronic obstructive pulmonary disease [19]. Furthermore, across the range of OSA severities previously studied, the absence of adverse respiratory effects was consistent, regardless of disease severity [18]. However, data on the effects of daridorexant in individuals with severe OSA are currently lacking. To address this evidence gap, the present trial was designed to evaluate the effect of daridorexant 50 mg on nighttime respiratory function and sleep in participants with severe OSA.

## Materials and methods

### Trial design

This was a randomized, double-blind, placebo-controlled, two-period, crossover pharmacology trial in participants with severe OSA and no insomnia conducted at one site (ASR Berlin, Germany) from July 2022 (first subject first visit) to February 2023 (last subject last visit). The trial protocol was approved by the German health authority (Bundesinstitut für Arzneimittel und Medizinprodukte, BfArM) and by the local Ethics committee (Ethik-Kommission des Landes Berlin, Berlin). The trial was conducted in accordance with the Declaration of Helsinki principles, International Council for Harmonization, Good Clinical Practice guidelines, and applicable regulations and laws. Each participant provided written informed consent prior to any screening procedures.

The trial design was similar to that used in a previous Phase 1 trial in participants with mild/moderate OSA [17]. Briefly, after a screening period, 16 participants were randomized in a 1:1 ratio to receive evening, oral doses of daridorexant (50 mg once daily) followed by a matching placebo or vice versa for five consecutive days with a 1–2-week washout period. The 5-day exposure duration was considered sufficient as steady-state exposure of daridorexant and its metabolites is reached after 3 days without relevant accumulation [20–22]. Eight-hour polysomnography (PSG) recordings were performed in each participant at the trial site at baseline (two screening visits), at Night 1 (after first dosing), and at Night 5 (after last dosing) of each period using MiniScreen PRO Viewer Software 5.21B R2 (Löwenstein Medical, Rheinland-Pfalz, Germany), as previously described [17]. Each PSG recording was attended by a sleep scientist and included electroencephalogram, electrooculogram, chin and leg electromyogram, measurement of respiratory efforts and nasal airflow, and pulse oximetry. PSG recordings were started within 1 hour of the individual's usual bedtime as determined by the participant’s self-reporting during the screening period. This approach was applied to avoid disrupting individual sleep habits. PSG recordings ended after 8 hours when 960 epochs of 30 sec had been recorded, i.e. time of 'lights on', to ensure standardization both within and between subjects, given the potential for inter-individual and night-to-night variability and to ensure at least 6 hours of technically adequate data. An identical set-up was used in the respiratory trials in participants with OSA with two products of the same class, suvorexant and lemborexant [23, 24].

In both trial periods, first and last dosing occurred at the trial site, while from Night 2 to Night 4, study treatments were self-administered at home. Trial treatments were administered at the trial site approximately 30 min before bedtime and the start of the PSG recording. While at home, participants were similarly instructed to take trial treatment approximately 30 min before bedtime and at least 2 h after the evening meal. Trial staff contacted participants by telephone daily to ensure compliance to treatment intake.

### Trial population

Eligible participants were males and females aged ≥18 years with severe OSA as per the American Academy of Sleep Medicine guidelines [25]. Severe intensity of OSA was confirmed during both screening night PSGs and was defined as an apnea/hypopnea index (AHI) ≥ 30 events/h [25]. During screening, participants had to report a usual bedtime between 21:30 and 00.30 and regular time in bed between 6 and 9 h.

All participants underwent a comprehensive medical history interview, including a detailed sleep anamnesis conducted by a sleep specialist. Participants were excluded if they had history of other respiratory disorders besides OSA or any known factor or disease with a potential impact on sleep, motor performance, or cognition (i.e. psychiatric disease, neurological disorder, insomnia disorder), or any clinically significant abnormality present on either or both screening night PSGs, including evidence of insomnia not explained by OSA, periodic limb movement disorder with arousal index ≥10/h, restless legs syndrome, circadian rhythm disorder, rapid eye movement (REM) behavior disorder, parasomnia including nightmare disorder, sleep terror disorder, and sleepwalking disorder. Participants were also excluded if they had used a continuous positive airway pressure (CPAP) device or a dental appliance within at least 2 months prior to first administration of trial treatment or if they were required to use either during the trial period.

### Endpoints

The primary and secondary endpoints were the treatment differences (daridorexant–placebo) for AHI and mean nocturnal oxygen saturation (SpO_2_) during total sleep time (TST), respectively, after multiple-dose administration (Night 5 [after five doses] i.e. at steady state). AHI was calculated by dividing the total number of apneas and hypopneas by TST (in min) and multiplying by 60 (i.e. average number of events per h of sleep). Apneas were defined as a pause in respiration ≥10 sec. Hypopneas were defined as a reduction of airflow by ≥30% for ≥10 sec accompanied by a decrease in SpO_2_ by ≥3% compared to corresponding data prior to the hypopnea event and/or the event was associated with an arousal. After placebo correction, a mean increase in AHI during TST ≥10 events/h [26] and a mean decrease in nocturnal SpO_2_ during TST ≤ –2% [24] after multiple dosing of daridorexant 50 mg were the minimum changes considered to be clinically meaningful negative effects in participants with severe OSA.

Exploratory endpoints related to respiratory function (after multiple-dose administration) included AHI and SpO_2_ during REM sleep and non-REM sleep including N1, N2, and N3 sleep stages, oxygen desaturation index (ODI; average number of desaturation episodes [defined as a decrease in the mean oxygen saturation of ≥3% over the last 120 sec lasting ≥10 sec] per h of sleep) during TST and the mean and lowest SpO_2_ during respiratory events. Exploratory events related to sleep characteristics included PSG-determined TST, latency to persistent sleep (LPS), wake after sleep onset (WASO), and awakenings. An awakening was defined as time spent awake for at least 15 s and scored in a 30 s window (i.e. epoch).

Safety and tolerability were assessed by clinical assessment of adverse events (AEs) and other safety measurements (vital signs, 12-lead electrocardiogram [ECG], physical examination, clinical chemistry, hematology).

### Statistical analysis

The trial was powered to evaluate whether daridorexant 50 mg at steady state produced a clinically meaningful negative effect on AHI (primary endpoint) and SpO_2_ (secondary endpoint) during TST, i.e. whether the treatment difference (daridorexant–placebo) was >10 events/h and < −2%, respectively, in participants with severe OSA.

Type I error was set to 5% one-sided for the primary and secondary endpoints. For the primary endpoint, the null hypothesis was rejected if the one-sided 95% confidence interval (CI) of the treatment difference was ≤10 events/h; for the secondary endpoint, it was rejected if the one-sided 95% CI of the treatment difference was ≥ −2%. Hierarchical testing procedure was applied to ensure a Type I error of 5% across these two endpoints.

For the primary and secondary endpoints, individual AHI and SpO_2_ values after Night 5 were evaluated in a linear mixed-effect model appropriate for a two-period crossover trial. The model contained treatment, period, and baseline as fixed effects, and subject as random effect. The one-sided 95% CI (which corresponds to 90% CI two-sided) for the least square mean (LSM) treatment difference (daridorexant–placebo) at Night 5 was used to test the null hypotheses. Exploratory endpoints were also evaluated by linear mixed-model effect analyzes as per the primary/secondary endpoint.

Results are reported as LSM difference and two-sided 90% CI, for treatment difference (daridorexant–placebo).

### Power

For the primary endpoint (AHI), assuming a standard deviation (SD) for individual treatment differences of 12.0 events/h, a true mean difference of 2.38 events/h [17], and a threshold of 10 events/h for a clinically meaningful effect of multiple-dose administration of daridorexant 50 mg, a total of 17 participants was needed to reach 80% power.

For the secondary endpoint (SpO_2_), assuming an SD for individual treatment differences of 1.05%, a true mean difference of 0.16% [17], and a threshold of −2% for a clinically meaningful effect of multiple-dose administration of daridorexant 50 mg, the power obtained with 17 participants was 99.9%.

As the variability of AHI and SpO_2_ is not well described in participants with severe OSA, as planned in the protocol, once 15 participants were enrolled, the baseline data were used to re-evaluate the initially determined number of participants. From this evaluation, a sample size of 11 was required to obtain 80% power and as planned in protocol, recruitment was considered complete once 16 participants were enrolled.

## Results

### Trial participants

Sixteen participants with severe OSA were randomized, completed the trial, and included in the all-treated analysis set. There were 15 male and 1 female participants, all White, with a mean age of 63.1 years (range 49, 78) and a mean body mass index of 30.7 kg/m^2^ (range 21.3, 36.9). At baseline, mean (range) AHI and nocturnal SpO_2_ during TST were 51.2 (30.8, 82.2) events/h and 92.1% (88.5, 94.3), respectively (Table 1).

**Table 1:** Effect of daridorexant 50 mg on nighttime respiration-related variables

	**Baseline (*N* = 16)**	**Multiple dosing for 5 days**		
		**Daridorexant (*N* = 16)**	**Placebo (*N* = 15)**	Treatment difference^*^
AHI (/h) during:				
TST	51.2 (17.0)	44.2 (37.4, 50.9)	47.9 (40.9, 54.9)	–3.7 (–11.7, 4.2)
Non-REM	52.2 (16.5)	45.1 (38.0, 52.3)	47.7 (40.3, 55.1)	–2.6 (–11.0, 5.8)
N1	71.0 (17.8)	66.1 (57.7, 74.4)	71.9 (63.3, 80.6)	–5.9 (–15.7, 4.0)
N2	49.1 (17.8)	41.7 (34.1, 49.2)	45.0 (37.2, 52.8)	–3.3 (–12.3, 5.6)
N3	22.2 (23.3)	19.1 (6.5, 31.6)	19.0 (5.9, 32.2)	0.0 (–13.4, 13.4)
REM	45.4 (23.1)	37.2 (29.7, 44.7)	45.4 (37.9, 53.0)	–8.2 (–13.7, –2.7)
No. apneas during TST	188.6 (161.8)	168.0 (124.4, 211.6)	166.7 (121.7, 211.8)	1.3 (–46.7, 49.2)
No. hypopneas during TST	149.2 (106.9)	142.3 (109.7, 175.0)	147.7 (113.9, 181.5)	–5.4 (–43.9, 33.2)
Mean duration of apneas, sec	24.3 (8.9)	24.9 (20.7, 29.1)	26.8 (22.3, 31.3)	1.9 (–6.8, 3.0)
Mean duration of hypopneas, sec	23.9 (4.5)	27.8 (25.0, 30.6)	25.3 (22.4, 28,1)	2.5 (0.1, 4.9)
Mean SpO_2_ (%) during:				
TST	92.1 (1.7)	92.3 (91.6, 92.9)	92.4 (91.8, 93.0)	–0.1 (–0.6, 0.4)
Non-REM	92.2 (1.5)	92.3 (91.7, 93.0)	92.5 (91.9, 93.1)	–0.2 (–0.6, 0.3)
N1	92.3 (1.6)	92.4 (91.8, 93.0)	92.5 (91.9, 93.1)	–0.1 (–0.7, 0.5)
N2	92.2 (1.5)	92.3 (91.7, 93.0)	92.6 (91.9, 93.2)	–0.2 (–0.7, 0.2)
N3	92.4 (1.8)	92.5 (91.7, 93.2)	92.6 (91.8, 93.3)	–0.1 (–0.9, 0.7)
REM	91.2 (3.0)	92.2 (91.3, 93.1)	91.7 (90.8, 92.6)	0.5 (–0.3, 1.3)
Awake time	93.2 (1.3)	92.8 (92.0, 93.6)	93.4 (92.5, 94.2)	–0.6 (–1.5, 0.4)
% TST with SpO_2_:				
<90%	15.4 (16.1)	13.5 (9.0, 18.0)	13.2 (8.6, 17.8)	0.3 (–4.3, 5.0)
<85%	4.9 (7.5)	5.8 (2.4, 9.2)	5.0 (1.6, 8.5)	0.8 (–0.9, 2.5)
<80%	1.5 (2.5)	2.1 (0.4, 3.8)	1.7 (0.0, 3.5)	0.4 (–0.3, 1.1)
ODI during TST (/h)	46.6 (18.3)	40.3 (33.6, 46.9)	42.2 (35.4, 49.1)	–1.9 (–9.8, 5.9)
Lowest SpO_2_ during respiratory events (%)	74.8 (9.1)	77.0 (74.2, 79.9)	76.5 (73.5, 79.4)	0.6 (–2.8, 3.9)
Mean SpO_2_ during respiratory events (%)	88.4 (3.0)	88.4 (87.4, 89.4)	88.7 (87.7, 89.8)	–0.3 (–1.3, 0.6)

Data are displayed as arithmetic mean (SD) for baseline values, LSM (95% CI) for treatment and LSM (90%) for treatment difference.

^*^Statistics were calculated from the linear mixed-effects model with treatment, period, and baseline as fixed effects and subject as random effect.

AHI, apnea/hypopnea index; CI, confidence interval; REM, rapid eye movement; SpO_2,_ peripheral oxygen saturation; TST, total sleep time.

### Effect of daridorexant on AHI and SpO_2_ during TST

**Figure 1:**
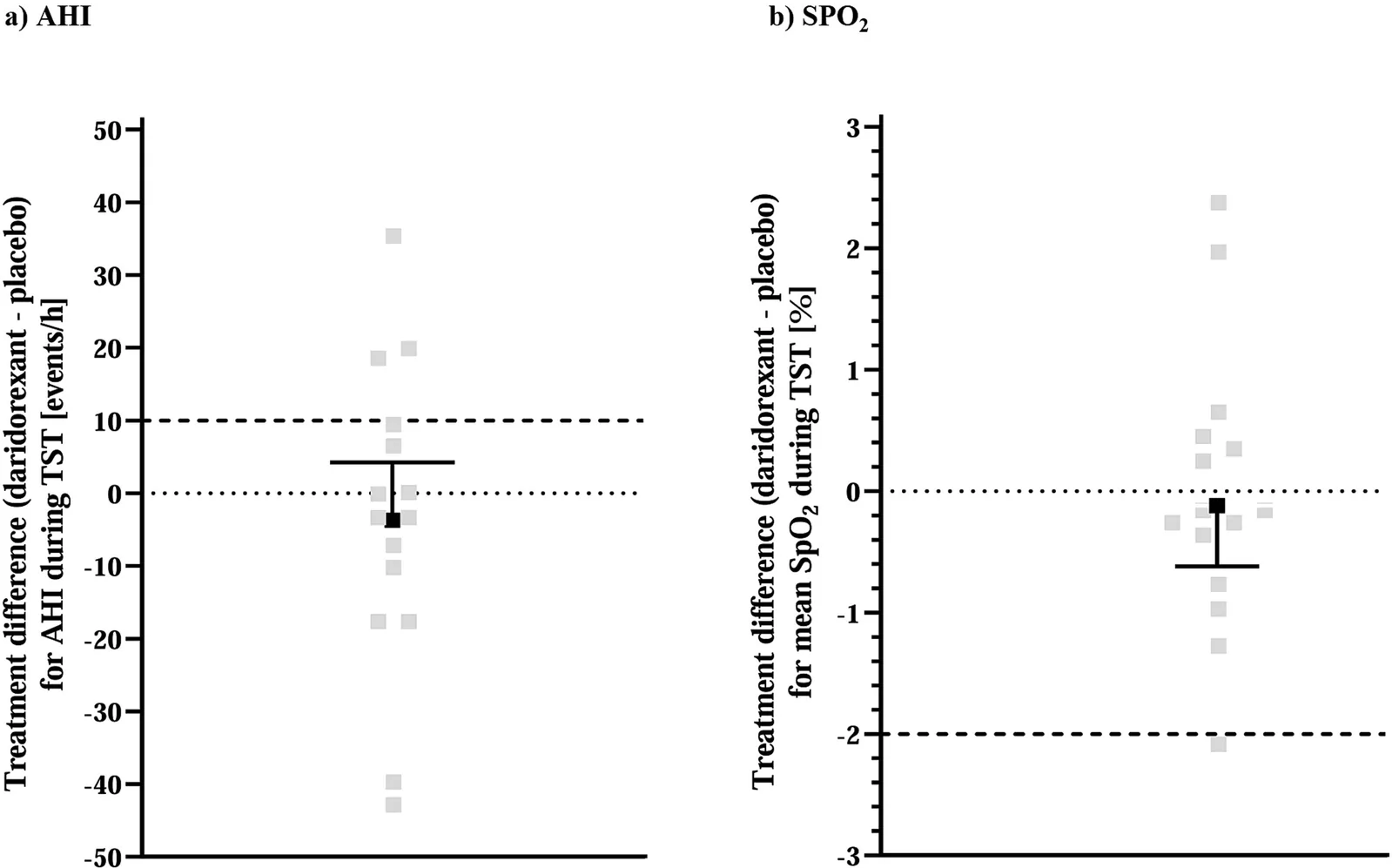
Treatment difference (daridorexant–Placebo) for (a) AHI and (b) SpO_2_ during TST after multiple-dose administration (*N* = 16). Data are provided as individual treatment differences (daridorexant–placebo) and LSM (+ 95% CI for AHI and -95% CI for SpO_2_; one-sided) for the treatment difference. **- - - -**: Threshold for a clinically meaningful negative effect of daridorexant. AHI, apnea/hypopnea index; CI, confidence interval; SpO_2,_ peripheral oxygen saturation; TST, total sleep time.

After multiple-dose administration of daridorexant 50 mg, the mean treatment difference (daridorexant–placebo) was −3.7 events/h (one-sided 95% CI ≤ 4.2) for AHI and − 0.1% (one-sided 95% CI ≥ −0.6) for nocturnal SpO_2_, during TST (Figure 1, Table 1). With the one-sided 95% CI <10 events/h for AHI and > −2% for SpO_2_ thresholds, no clinically meaningful negative effects were observed. There was, nonetheless, some heterogeneity in treatment response in this small sample (min, max; AHI: −43.0, 34.2 events/h; SpO_2_: −2.1, 2.3%). Three participants presented a marked treatment difference in AHI, whether positive or negative. One participant had a treatment difference of +34.2 events/h (AHI at two screening visits: 70.6 events/h and 49 events/visit; under daridorexant: 71.8 events/h; under placebo: 37.6 events/h), with minimal impact on SpO₂ (treatment difference -0.3%). A second participant had a treatment difference of -39.9 events/h (screening AHI: 31.4 and 30.2 events/h; under daridorexant: 8.6 events/h; under placebo: 48.5 events/h) while a third had a treatment difference of -43.0 events/h (screening AHI: 44.5 and 50.5 events/h; under daridorexant: 5.4 events/h; under placebo: 48.4 events/h); these decreases were associated with longer TST and reduced sleep in the supine position. Also, no treatment difference (daridorexant–placebo) was observed after multiple administration for other respiratory safety indices such as mean duration of apneas and hypopneas, time spent with SpO_2_ < 90%, <85%, and < 80%, ODI and the lowest and mean SpO_2_ during respiratory events (Table 1).

### Effect of daridorexant on AHI and SpO_2_ during REM and non-REM sleep phases

**Figure 2:**
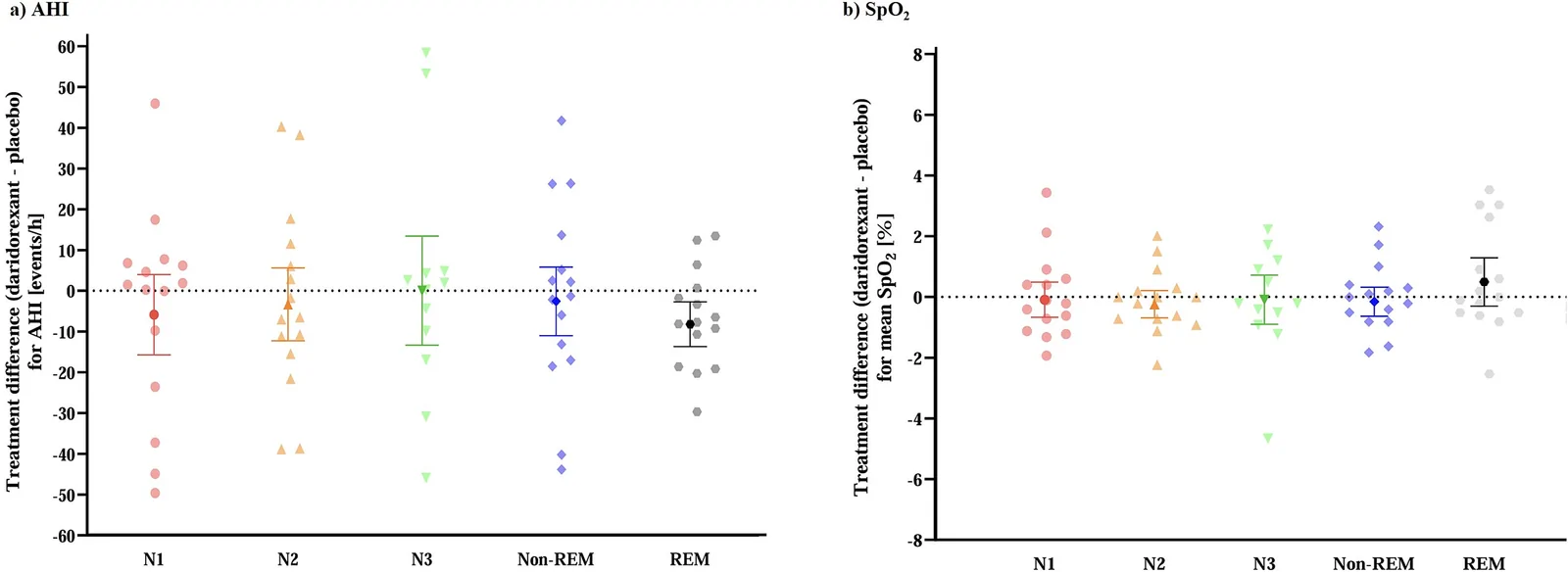
Treatment difference (daridorexant–placebo) for (a) AHI and (b) SpO_2_ during non-REM and REM sleep phases after multiple dose administration (*N* = 16). Data are provided as individual treatment differences (daridorexant–placebo) and LSM (± 90% CI for both AHI and SpO_2_; two-sided) for the treatment difference. AHI, apnea/hypopnea index; CI, confidence interval; REM, rapid eye movement; SpO_2,_ oxygen saturation.

After multiple-dose administration, no treatment difference (daridorexant–placebo) was observed for AHI during the non-REM sleep phase, including sleep stages N1 to N3 (Figure 2, Table 1). AHI during REM sleep after multiple-dose administration of daridorexant (LSM: 37.2 events/h [95% CI: 29.7, 44.7]) was numerically lower than after placebo (LSM: 45.4 events/h [95% CI: 37.9, 53.0]; LSM difference − 8.2 events/h [90% CI: −13.7, −2.7]). Accordingly, no treatment difference (daridorexant–placebo) was observed for SpO_2_ at any time during the night (non-REM [including sleep stages N1 to N3] and REM sleep phases) (Figure 2, Table 1).

### Effect of daridorexant on sleep parameters

**Figure 3:**
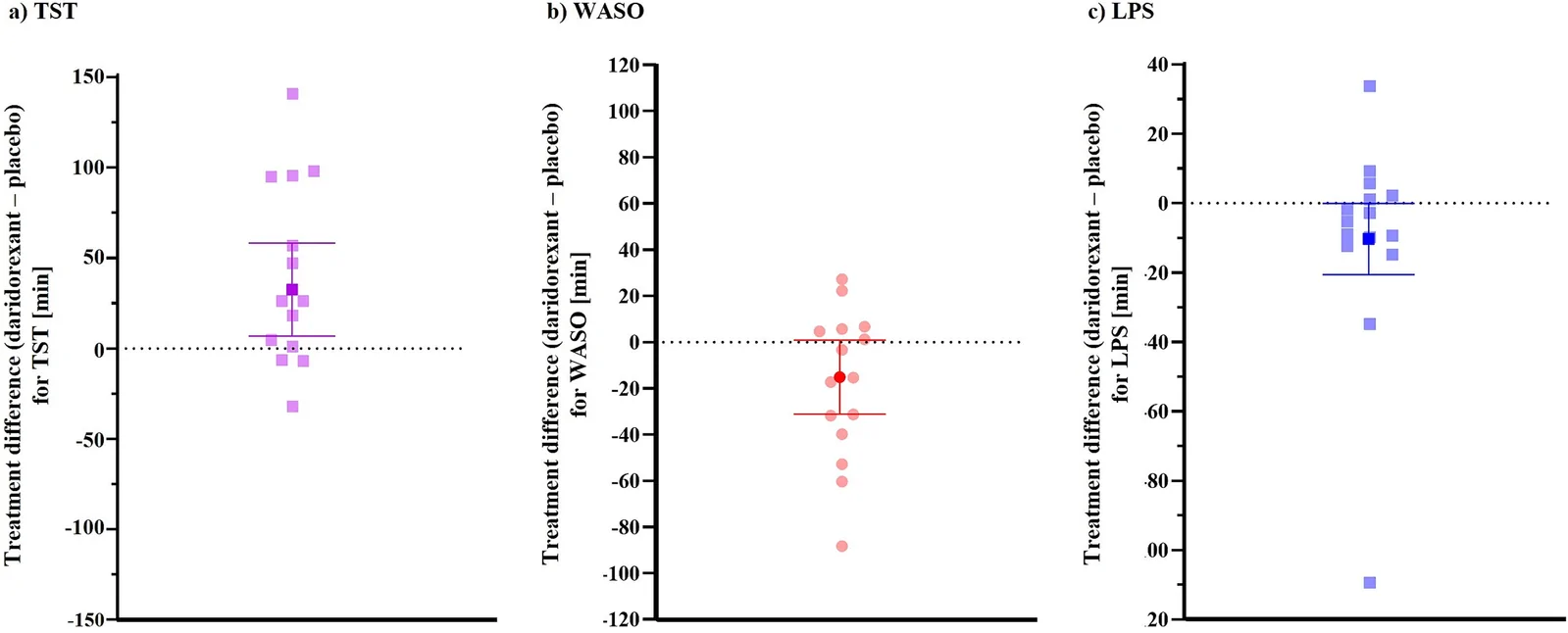
Treatment difference (daridorexant–placebo) for sleep parameters after multiple dose administration (*N* = 16). Data are provided as individual treatment differences (daridorexant–placebo) and LSM (± 90% CI two-sided) for the treatment difference for (a) TST, (b) WASO, and (c) LPS after multiple dose administration. LPS, latency to persistent sleep; TST, total sleep time; WASO, wake after sleep onset.

After multiple dosing, daridorexant, when compared with placebo, prolonged mean TST by 32.5 min (90% CI: 6.9, 58.2) (Figure 3, Table 2). The increase in TST was associated with a shorter LPS (−10.3 min [90% CI: −20.6, −0.02]) and a trend towards a reduced WASO (−15.2 min [90% CI: −31.2, 0.9]). The prolongation of TST was mainly due to an increased duration of REM sleep (treatment difference of +28.8 min [90% CI 16.3, 41.2).

**Table 2:** Effect of daridorexant 50 mg on sleep parameters

	**Baseline (*N* = 16)**	**Multiple dosing for 5 days (*N* = 16)**		
		**Daridorexant**	**Placebo**	Treatment difference^*^
TST, min	393.6 (56.0)	424.2 (399.0, 449.4)	391.7 (365.7, 417.7)	32.5 (6.9, 58.2)
LPS, min	23.3 (21.7)	11.9 (3.2, 20.7)	22.2 (13.2, 31.2)	–10.3 (–20.6, –0.0)
WASO, min	60.2 (40.1)	41.4 (15.7, 67.1)	56.5 (30.4, 82.6)	–15.2 (–31.2, 0.9)
Sleep stages, duration, min				
N1	96.0 (41.1)	84.2 (67.5, 100.9)	80.2 (63.1, 97.2)	4.0 (–8.1, 16.1)
N2	197.6 (49.7)	216.3 (202.3, 230.3)	212.6 (198.1, 227.1)	3.7 (–12.5, 19.8)
N3	40.8 (32.3)	41.8 (28.5, 55.1)	46.8 (33.1, 60.5)	–5.0 (–20.2, 10.2)
REM	59.2 (22.4)	81.9 (69.7, 94.1)	53.1 (40.6, 65.7)	28.8 (16.3, 41.2)
Awakenings				
Number of awakenings	17.5 (7.3)	13.7 (10.9, 16.4)	14.3 (11.4, 17.1)	–0.6 (–3.9, 2.7)
Mean duration of awakening, min	4.8 (3.4)	4.0 (1.8, 6.2)	6.3 (4.0, 8.5)	–2.3 (–3.9, –0.7)
Longest duration of awakening, min	29.5 (22.7)	23.3 (4.7, 41.8)	42.0 (23.1, 60.9)	–18.7 (–31.1, –6.4)
Arousal index, total, /h	32.0 (17.8)	31.3 (25.3, 37.4)	31.3 (25.1, 37.5)	0.04 (–5.9, 6.0)
Time spent in supine position, % TST	40.2 (30.2)	37.6 (29.6, 45.5)	36.3 (28.1, 44.5)	1.3 (–7.7, 10.3)

Data are displayed as arithmetic mean (SD) for baseline values, LSM (95% CI) for treatment and LSM (90%) for treatment difference.

^*^Statistics were calculated from the linear mixed-effects model with treatment, period, and baseline as fixed effects and subject as random effect.

CI, confidence interval; LPS, latency to persistent sleep; TST, total sleep time; WASO, wake after sleep onset.

After multiple dosing of daridorexant 50 mg, the number of awakenings was similar to placebo (treatment difference − 0.6 [90% CI: −3.9, 2.7]). However, compared to placebo, the duration of the longest awakening was shortened by 18.7 min (90% CI: –31.1, –6.4) and the mean duration of awakenings was shortened by 2.3 min (90% CI: –3.9, –0.7) (Table 2).

Mean arousal index and time spent in supine position remained largely unchanged from baseline, with no meaningful differences between daridorexant and placebo (Table 2).

### Safety of daridorexant in participants with severe OSA

A total of four treatment-emergent AEs were reported by three different participants (three AEs with daridorexant [2 participants], one with placebo [1 participant]) (Table 3). All AEs were of mild intensity and resolved, and none were related to respiratory function. In addition, two AEs of nasopharyngitis were reported but were considered not treatment-emergent. There were no deaths, serious AEs or discontinuations due to AEs. No changes from baseline were observed for ECG, vital signs, or clinical laboratory variables.

**Table 3:** Summary of treatment-emergent AEs

	**Daridorexant 50 mg (*N* = 16)**	**Placebo (*N* = 16)**
Participants with at least one AE, n (%)	2 (12.5)	1 (6.3)
AEs, n (%)		
Somnolence	1 (6.3)	1 (6.3)
Headache	1 (6.3)	–
Abnormal dreams	1 (6.3)	–

AE, adverse event.

## Discussion

The primary aim of this pharmacological trial was to evaluate the effects of daridorexant on nighttime respiratory function in participants with severe OSA in order to indicate whether daridorexant can be safely recommended for patients with insomnia who may also have underlying OSA, whether diagnosed or undiagnosed. This trial shows that daridorexant 50 mg, the highest approved dose, is well-tolerated and has no clinically meaningful negative effect on nighttime respiratory function in this patient population. Despite the absence of insomnia disorder in this group, treatment with daridorexant led to improvements in objective (PSG-determined) sleep parameters compared with placebo.

The findings of this trial in individuals with severe OSA are consistent with previous results observed in individuals with mild/moderate OSA without insomnia utilizing a similar trial design [17, 18]. Moreover, in line with the established safety profile of daridorexant in the insomnia clinical trials [27], as well as the known safety profile of DORAs as a class, daridorexant 50 mg was generally safe and well-tolerated, based on AEs, vital signs, ECG and laboratory data. Importantly, no evidence of respiratory depressant effects was observed following multiple dosing with daridorexant. Similarly, lemborexant (another DORA) has been reported not to be associated with adverse respiratory effects in participants with severe OSA, as assessed by AHI and SpO_2_ [23]. Given that in OSA, the frequency and duration of apneas and hypopneas often increase during REM sleep compared to non-REM sleep [28], it is noteworthy that, although the trial was not powered for this endpoint, a reduction in AHI during REM sleep was observed following multiple dosing with daridorexant versus placebo (LSM treatment difference − 8.2 events/h), while no difference was observed for non-REM sleep, including sleep stages N1 to N3.

Although AHI during TST is commonly used for the diagnosis of OSA, it may not fully capture the breadth of nighttime respiratory safety. To strengthen the evidence supporting the safety of daridorexant on nighttime respiration, we assessed additional parameters not previously reported for DORAs in severe OSA, including the mean duration of apneas and hypopneas, the oxygen desaturation index and the lowest SpO_2_ recorded during respiratory events. Overall, the results are consistent with the primary and secondary outcomes and further support the absence of any negative effect of daridorexant on sleep-disordered breathing. These results also align with those obtained in individuals with less severe forms of OSA treated with daridorexant [17].

Notably, in OSA patients without insomnia, multiple administration of daridorexant 50 mg resulted in nominal improvements in objective sleep measures, with mean TST prolonged by 32.5 min vs placebo. This increase in TST may be explained by a trend towards reduced WASO (decreased by approximately 15 min) as well as a shorter LPS (decreased by approximately 10 min). Daridorexant also reduced both the mean and longest duration of awakenings while maintaining the total number of nocturnal awakenings versus placebo. These findings are consistent with previous wake bout analyzes [30], align with the observed trend towards a reduced WASO and suggest improved sleep continuity. The potential impact of these changes on daytime sleepiness and related symptoms warrants investigation in future studies. These results are again consistent with previous findings in subjects with mild/moderate OSA [17], as well as with those suggested by the post hoc analysis of the Phase 3 daridorexant trials in patients with insomnia disorder plus mild OSA [31], and together suggest that daridorexant can stabilize sleep variables in subjects with severe OSA.

This trial has several strengths and limitations that should be considered when interpreting the findings. Severe OSA is defined as AHI ≥30 events/h [25]. The trial population reflected a broad spectrum of severe OSA phenotypes with baseline AHI values ranging from 31 to 82 events/h. Conducting the trial at a single center optimized the homogeneity and consistency of assessments; however this may also limit the external validity and generalizability of the results. The trial size and treatment duration were adequate to address the primary and secondary hypotheses but nonetheless represent limitations. Due to the inherent inclusion criteria, the studied population may not fully represent the broader severe OSA population typically encountered in clinical practice. In particular, the male-to-female ratio (15:1) deviates markedly from the real-world prevalence ratio (~2.5:1) [32]. In addition, no specific criteria were applied to restrict subtypes of OSA, such as positional OSA. While this helps enhance the generalizability of the findings, this may have contributed to the heterogeneity observed in the treatment difference (daridorexant vs placebo) for AHI, which we postulate is due to inter-night variability. AHI can fluctuate significantly from night to night in the same individual due to factors such as body position, sleep stage, sleep environment, and other additional sleep-affecting medications [33] and should be accounted for when designing future trials. Indeed, three participants were notable outliers, with treatment differences in AHI ranging from +34.2 to −43.0 events/h. Importantly, these changes were attributable to night-to-night fluctuation, longer TST, and reduced sleep in the supine position with no evidence that daridorexant per se increased AHI, supporting its respiratory safety in patients with severe OSA. In addition, at baseline (mean of the two PSG assessments), while mean TST was 398 min (~6.5 h), two participants had TST < 5 h, likely reflecting the “first-night” effect in sleep laboratory conditions [29]. One showed an increased TST between the two screening PSG assessments, while the other had poor sleep due to OSA, with TST similar to baseline under placebo but increased by 140 min under daridorexant after repeated dosing. In this trial, daridorexant was evaluated in participants with severe OSA who were not using CPAP, thus representing a population reflective of undiagnosed OSA. Subjects with comorbid insomnia were excluded to ensure sufficient TST for reliable measurement of AHI.

## Conclusions

Daridorexant has previously been shown to have no deleterious effects on nighttime respiratory function in participants with mild/moderate OSA without insomnia. The current findings extend this evidence by indicating that multiple doses of daridorexant 50 mg are also safe and well-tolerated in participants with severe OSA not using CPAP and not having insomnia, with no increase in the frequency of apnea/hypopnea events nor in oxygen desaturation. In addition, despite the absence of coexisting insomnia disorder, data suggest that daridorexant may improve sleep in this population.

Thus, these findings indicate that daridorexant can be recommended for patients with OSA irrespective of its severity. Further studies might assess whether daridorexant helps in improving compliance with mechanical standard therapies such as positive airway pressure or mandibular protrusion devices.

## Data Availability

In addition to Idorsia’s existing clinical trial disclosure activities, the company is committed to implementing the Principles for Responsible Clinical Trial Data Sharing jointly issued by the European Federation of Pharmaceutical Industries and Associations (EFPIA) and the Pharmaceutical Research and Manufacturers of America (PhRMA). Requests for data sharing, of any level, can be directed to clinical-trials-disclosure@idorsia.com for medical and scientific evaluation.
